# A Boosting SAR Image Despeckling Method Based on Non-Local Weighted Group Low-Rank Representation

**DOI:** 10.3390/s18103448

**Published:** 2018-10-13

**Authors:** Jing Fang, Shaohai Hu, Xiaole Ma

**Affiliations:** 1 Institute of Information Science, Beijing Jiaotong University, Beijing 100044, China; maxiaole@bjtu.edu.cn; 2 Shandong Province Key Laboratory of Medical Physics and Image Processing Technology, School of Physics and Electronics, Shandong Normal University, Jinan 250014, China

**Keywords:** synthetic aperture radar, low-rank representation, non-local, speckle noise reduction, boosting

## Abstract

In this paper, we propose a boosting synthetic aperture radar (SAR) image despeckling method based on non-local weighted group low-rank representation (WGLRR). The spatial structure information of SAR images leads to the similarity of the patches. Furthermore, the data matrix grouped by the similar patches within the noise-free SAR image is often low-rank. Based on this, we use low-rank representation (LRR) to recover the noise-free group data matrix. To maintain the fidelity of the recovered image, we integrate the corrupted probability of each pixel into the group LRR model as a weight to constrain the fidelity of recovered noise-free patches. Each single patch might belong to several groups, so different estimations of each patch are aggregated with a weighted averaging procedure. The residual image contains signal leftovers due to the imperfect denoising, so we strengthen the signal by leveraging on the availability of the denoised image to suppress noise further. Experimental results on simulated and actual SAR images show the superior performance of the proposed method in terms of objective indicators and of perceived image quality.

## 1. Introduction

Synthetic aperture radar (SAR) remote sensing has been extensively applied in military and civil fields because of the all-day, all-weather acquisition capability. However, being acquired via coherent imaging, the SAR images are intrinsically associated with a signal-dependent granular noise called speckle [1]. The existence of speckle degrades the appearance of images which may affect the performance in many applications such as target detection, terrain classification, etc. Thus, despeckling is an important preprocessing step for a number of applications [1]. Such preprocessing, however, should be carefully designed to suppress most of the speckle in homogeneous regions and preserve textures and region boundaries, while avoiding the introduction of filtering artifacts.

Early works on despeckling were performed in the spatial domain. The Lee filter [2] is reportedly the first model-based despeckling filter. It is thoroughly developed in [3] and reviewed in [4] together with the sigma filter. The Lee refined filter [5] uses the local gradient to estimate the orientation of the edge boundaries which is noisy in the Lee filter. The Frost filter [6] starts from a model of the coherent imaging system and constructs an auto-correlation function from local statistics. The noise-free image can be estimated by solving the function with the linear minimum mean square error (LMMSE) estimator. Different filter sizes greatly affect the quality of the estimated images. Although classical spatial filters can realize the result of speckle removal, they can lead to blurring effects and defects in detail preservation, which has a bad influence on the subsequent image processing. As a popular denoising method, total variation (TV) regularization is used in the multiplicative noise by some scholars [7,8]. In such a method, denoising is achieved by the minimization of a cost function based on the intensity or the logarithm of the intensity. However, many methods have been validated on simulated data and less used in actual SAR images [1].

Filtering in the transform domain has been extensively used during the last twenty years, such as wavelet transform [9,10,11,12], principal component analysis [13,14] and sparse representation [15,16,17]. Wavelet shrinkage can be readily applied to SAR despeckling after a homomorphic transformation. Each wavelet subband is associated to a speckle contribution that may be exactly measured. Classical hard-thresholding and soft-thresholding methods are applied in [9,10], respectively. Some scholars [18,19,20] have performed a statistical Bayesian estimation to optimize the shrinkage parameter. In [21], undecimated wavelet and Maximum A Posteriori (MAP) estimation are used for despeckling. Argenti [22] and Bianchi [23] extended the despeckling to the nonsubsampled contourlet transform domain. However, the decomposition of subbands in the transform domain may cause unwanted artifacts. Nonlocal (NL) filtering has been successfully applied to SAR images denoising in the wavelet domain [24,25,26]. NL filtering is applied by substituting the Euclidean distance with a probabilistic measure according to the pdf of SAR data, which provided us with the new research direction.

Recently, low-rank representation (LRR) has been extensively used in image processing [27,28] and image restoration [29,30,31]. Meanwhile, the similarity of the ground objects leads to LRR of SAR images [32,33,34]. Considering the severe speckle noise in SAR images, it is a challenge to decompose the corrupted matrix into a noise matrix and a low-rank matrix of the noise-free image. In this paper, we propose a boosting SAR image despeckling method based on non-local weighted group low-rank representation (WGLRR). By logarithmic transformation, the multiplicative noise becomes additive. Grouping the similar patches by an ad hoc measure to form groups of similar image blocks, we use the low rank representation model for speckle noise reduction of the similar image blocks. To ensure the fidelity of the recovered image, the corrupted probability of each pixel is added to the LRR model as a weight to regularize the reconstruction error term. Each single patch may belong to several groups, so we aggregate the different estimates by different weights depending on the rank of each group data matrix to obtain the denoised result. To suppress the speckle further and improve the despeckling SAR image, one boosting recursive method [35] is finally adopted.

The rest of the paper is organized as follows. In Section 2, we present the model of the noisy signal. Then, in Section 3, starting from the logarithmic model, the proposed method is rigorously described. The compared experiments on simulated and real SAR images are conducted in Section 4. Finally, Section 5 concludes this paper.

## 2. Models of Noisy Signal

Despeckling filters aim at estimating the noise-free radar reflectivity from the observed noisy SAR images under a statistical signal processing perspective. Under the hypothesis of fully developed speckle, the observed backscattered signal can be expressed as [36]:

(1) z=xu

where *x* is a possibly auto-correlated random process and represents the noise-free reflectivity; *u* is a possibly auto-correlated stationary random process, independent of *x*, representing the speckle fading term; and *z* is the observed noisy image. All the quantities in Equation (1) may refer to either intensity or amplitude as well as to single-look or multi-look images. It is well established that the fully developed speckle follows the Gamma distribution [36]

(2) $$p_{v}\left(u\right)=\frac{L^{L}u^{L-1}}{\Gamma (L)}exp\left(-uL\right),\;\;u\geq 0$$

where *L* is the equivalent number of looks (ENL) and Γ() is the Gamma function. Taking the logarithm of the observed data, we have

(3) ln(z)=ln(x)+ln(u)

where ln(u) is a signal-independent additive noise. The mean and variance of ln(u) are related to the ENL.

(4) $$E\left[ln\left(u\right)\right]=\psi^{\left(0\right)}\left(L\right)-ln\left(L\right)$$

(5) $$var\left[ln\left(u\right)\right]=\psi^{\left(1\right)}\left(L\right)$$

where $\psi^{\left(m\right)}\left(L\right)$ is the polygamma function of order *m*. Since an unbiased estimation in the log-domain is mapped onto a biased estimation in the spatial domain, we perform the bias correction [37] to Equation (3),

(6) $$z^{\left(ln\right)}=ln\left(z\right)-\psi^{\left(0\right)}\left(L\right)+ln\left(L\right)$$

The following filtering process will work on the bias-corrected log-intensity data.

## 3. The Proposed Method

### 3.1. Block Similarity Measure

For ease of presentation, let Y denote the bias-corrected log-intensity data defined as

(7) Y=X+E

where X is the noise-free matrix, and E is the noise matrix.

Suppose that the size of X is $N_{1}\times N_{2}$. We extract sliding patches of size $\sqrt{M}\times \sqrt{M}$ from X. The number of patches is

(8) $$N=\left(\left\lceil \frac{N_{1}-\sqrt{M}}{SL^{\left(p\right)}}\right\rceil +1\right)\left(\left\lceil \frac{N_{2}-\sqrt{M}}{SL^{\left(p\right)}}\right\rceil +1\right)$$

where $SL^{\left(p\right)}$ is the step length. Let $y_{i}={\left[y_{1},y_{2},\cdots ,y_{M}\right]}^{T}$ be the column stacked version of these patches. All of the patches form a data matrix $\overset{\wedge }{Y}=\left(y_{1},y_{2},\cdots ,y_{N}\right)\in {ℜ}^{M\times N}$. It is well known that self-similarity is abundant in SAR images. For a given reference patch $y_{i}$, we use the block similarity measure (BSM) instead of the Euclidean distance as the similarity measurement which is more appropriate for SAR images. Inspired by the literature [24], we define the block similarity measure as

(9) $$d\left[y_{i},y_{j}\right]=-ln\left\{\prod_{n}p[y(i+n),y(j+n)]|x\left(i+n\right)=x\left(j+n\right)\right\}$$

where p(·) indicates a probability density function. Since, for an *L*-look amplitude, SAR image speckle can be modeled by a square root gamma distribution with order *L*, Equation (9) can be expressed as

(10) $$d\left[y_{i},y_{j}\right]=-ln\left\{\prod_{n}4L\frac{\Gamma (2L-1)}{\Gamma^{2}\left(L\right)}\times {\left[\frac{y(i+n)y(j+n)}{y^{2}\left(i+n\right)+y^{2}\left(j+n\right)}\right]}^{2L-1}\right\}$$

where *L* is the equivalent number of looks and Γ() is the Gamma function. According to the properties of logarithmic operation, Equation (10) can be expressed as

(11) $$\begin{array}{c} d\left[y_{i},y_{j}\right]=-ln\left[4L\frac{\Gamma (2L-1)}{\Gamma^{2}\left(L\right)}\right]-\sum_{n}ln{\left[\frac{y(i+n)y(j+n)}{y^{2}\left(i+n\right)+y^{2}\left(j+n\right)}\right]}^{2L-1}\\ =-ln\left[4L\frac{\Gamma (2L-1)}{\Gamma^{2}\left(L\right)}\right]+\left(2L-1\right)\sum_{n}ln\left[\frac{y(i+n)}{y(j+n)}+\frac{y(j+n)}{y(i+n)}\right] \end{array}$$

The first item is a constant, so the BSM can be approximated as

(12) $$d\left[y_{i},y_{j}\right]=\left(2L-1\right)\sum_{n}ln\left[\frac{y(i+n)}{y(j+n)}+\frac{y(j+n)}{y(i+n)}\right]$$

The smaller the *d*-distance is, the more similar $y_{i}$ and $y_{j}$ are. Grouping the pixels with similar local spatial structures in the local window, we can obtain the data matrix

(13) $$Y_{i}=\left(y_{i},y_{i,1},y_{i,2},\cdots ,y_{i,p}\right)\in {ℜ}^{M\times (p+1)}$$

where p≤N. Additionally, there are *p*-most similar patches of $y_{i}$.

Considering the added model of Equation (7), we have

(14) $$Y_{i}=X_{i}+E_{i}$$

where $X_{i}$ is the noise-free group matrix and $E_{i}$ is the noise matrix.

### 3.2. Weighted Group Low-Rank Representation Model

A conventional low-rank representation approach to recover the noise-free matrix from Equation (14) is to solve the following optimization problem

(15) $${\hat{X}}_{i}=\underset{X_{i}}{min}rank\left(X_{i}\right)+\lambda {∥E_{i}∥}_{F}^{2},\;\;s.t.\;\;Y_{i}=X_{i}+E_{i}$$

where λ is a parameter whose value is more than zero. ${∥\cdot ∥}_{F}^{2}$ indicates the squared Frobenius norm used for modeling the noise. Considering that Equation (15) does not make full use of the spatial structure information of the image, we improve the LRR model according to the statistics of SAR images.

The seriously corrupted pixels in SAR images will vary greatly in intensity from most or all of their neighboring pixels which is similar to impulse noise. Thus, we use the local image rank-ordered absolute differences (ROAD) statistic [38] to identify the speckle noisy pixels from a noisy image.

Let $u=(u_{1},u_{2})$ be the location of the pixel under consideration. The set of points in a 3×3 neighborhood centered at u is denoted as

(16) $$\Omega_{u}:=\left\{u+(i,j):-1\leq i,j\leq 1\right\}$$

For each point $r\in \Omega_{u}$, the absolute difference of the pixels between $q_{u}$ and $q_{v}$ is defined as

(17) $$d_{u,v}=\left|q_{u}-q_{v}\right|$$

**Figure 1:**
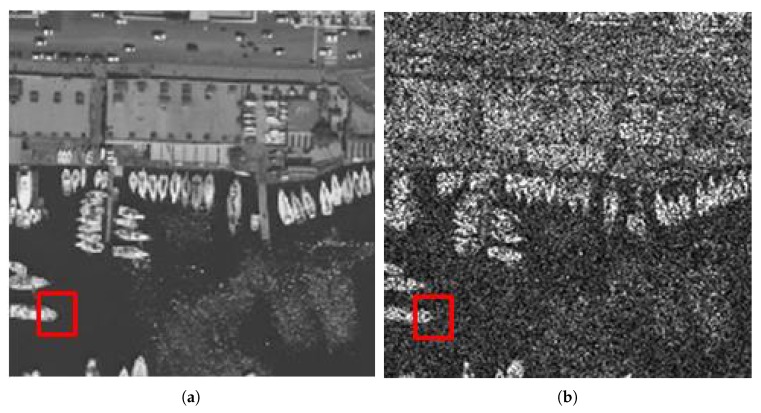
Naples image: (**a**) original image; and (**b**) noisy image corrupted by one-look speckle.

**Figure 2:**
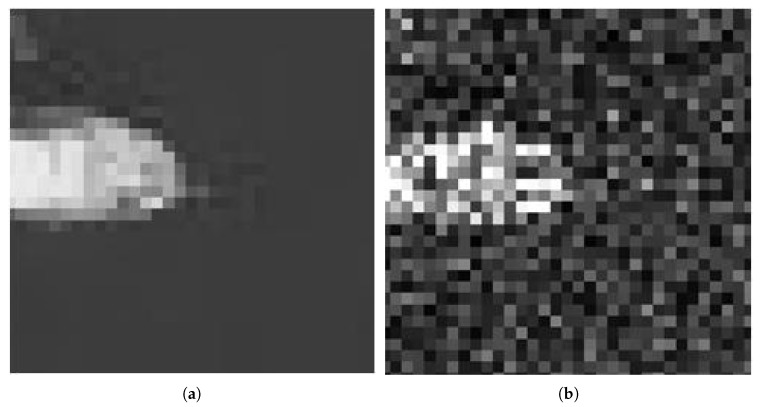
Zoom of a red rectangle area in Figure 1: (**a**) original image; and (**b**) noisy image corrupted by one-look speckle.

We sort all the absolute difference and select the *m*th smallest one of $d_{u,v}$ denoted as $d_{m}$. Then, the ROAD of pixel $q_{u}$ is defined as

(18) $$ROAD_{s}\left(q_{u}\right)=\sum_{m=1}^{s}d_{m}$$

where 2≤s≤7. In this paper, we set s=4. That is, we measure how close the $q_{u}$ is to its four most similar neighbors by the $ROAD_{4}\left(q_{u}\right)$. Ideally, we want the ROAD to be very high for speckle noise pixels while much lower for uncorrupted pixels. For illustrative purposes, we choose an aerial photo of the city of Naples [25] which shows a better similarity to SAR images in terms of scene structure. Figure 1 shows the original Naples image and the noisy image corrupted by one-look speckle. Figure 2 shows examples from the tested image comparing the enlarged noisy pixels to the original pixels which are marked by the red rectangle. Despite the enlarged area being part of an edge, it has neighbors of similar intensity which leads to a significantly lower ROAD value.

For example, we select a typical edge pixel with coordinate (50,166) in the red rectangle area. Then, the original neighborhood is shown as:

$$\left(\begin{array}{ccc} 156 & 227 & 233\\ 56 & 116 & 198\\ 65 & 62 & 65 \end{array}\right)$$

The absolute difference can be calculated as:

$$\left(\begin{array}{ccc} 40 & 111 & 117\\ 60 & - & 82\\ 51 & 54 & 51 \end{array}\right)$$

Selecting the four smallest absolute difference, the ROAD can be denoted as:

$$ROAD_{original}=\sum_{i=1}^{4}r_{i}=40+51++51+54=196$$

Similarly, the ROAD with the same coordinate in the noisy image can be calculated as:

$$ROAD_{noisy}=\sum_{i=1}^{4}r_{i}=21.1774+59.1651+114.9567+160.0594=355.3586$$

From the example above, we can see that the noise pixels often have much higher mean ROAD values than the uncorrupted pixels.

After calculating the ROAD of pixel $q_{u}$, we can express the final corrupted probability as

(19) $$p_{u}=1-exp\left(-0.01\cdot ROAD\left(q_{u}\right)\right)$$

Aggregating all the corrupted probability of each pixel, we can obtain the low-rank representation model:

(20) $${\hat{X}}_{i}=\underset{X_{i}}{min}rank\left(X_{i}\right)+\lambda {∥E_{i}∥}_{F}^{2},\;\;s.t.\;\;P_{i}\circ Y_{i}=P_{i}\circ X_{i}+E_{i}$$

where $P_{i}$ is composed of all the corrupted probability of pixels in the data matrix $Y_{i}$ and “∘” denotes multiplying the two matrices element-by-element. The constraints ensure a smaller error to the pixel with smaller corrupted probability which can better preserve the fidelity of the real noise-free image.

Solving the above optimization problem is not an easy work because of the discrete nature of the rank function. Wright et al. showed that we can recover the low-rank matrix by solving the following convex optimization problem [27]:

(21) $${\hat{X}}_{i}=\underset{X_{i}}{min}{∥X_{i}∥}_{*}+\lambda {∥E_{i}∥}_{F}^{2},\;\;s.t.\;\;P_{i}\circ Y_{i}=P_{i}\circ X_{i}+E_{i}$$

where ${∥\cdot ∥}_{*}$ denotes the nuclear norm of a matrix which can be calculated by the sum of its singular values. The recovery of matrix $X_{i}$ can be carried out by the augmented Lagrange multipliers (ALM) [39]:

(22) $$\begin{array}{c} L\left(X_{i},E_{i},R_{i},\beta \right)={∥X_{i}∥}_{*}+\lambda {∥E_{i}∥}_{F}^{2}+\langle R_{i},P_{i}\circ Y_{i}-\left(P_{i}\circ X_{i}+E_{i}\right)\rangle +\frac{\beta }{2}{∥P_{i}\circ Y_{i}-\left(P_{i}\circ X_{i}+E_{i}\right)∥}_{F}^{2}\\ \;\;\;\;\;\;\;\;\;\;\;\;\;\;\;\;\;\;\;\;\;\;\;={∥X_{i}∥}_{*}+\lambda {∥E_{i}∥}_{F}^{2}+\frac{\beta }{2}{∥P_{i}\circ Y_{i}-\left(P_{i}\circ X_{i}+E_{i}\right)+\frac{R_{i}}{\beta }∥}_{F}^{2}-\frac{1}{2\beta }{∥R_{i}∥}_{F}^{2} \end{array}$$

where β is a positive scalar so that the objective function is only perturbed slightly and $R_{i}$ is a Lagrange multiplier which is used to remove the equality constraint. $\langle \;\cdot \;\;,\;\;\cdot \rangle$ is the inner product.

Usually, it is difficult to minimize $L(X_{i},E_{i},R_{i},\beta )$ with respect to $X_{i}$ and $E_{i}$ simultaneously, so we resolve this problem by decomposing the minimization of Equation (22) into two subproblems.

(23) $$X_{i}^{k+1}=arg\underset{X_{i}}{min}L\left(X_{i},E_{i}^{k},R_{i}^{k},\beta \right)$$

(24) $$E_{i}^{k+1}=arg\underset{E_{i}}{min}L\left(X_{i}^{k+1},E_{i}^{},R_{i}^{k},\beta \right)$$

The subproblem in Equation (23) is equivalent to

(25) $$X_{i}^{k+1}=arg\underset{X_{i}}{min}{∥X_{i}∥}_{*}+\frac{\beta^{k}}{2}{∥P_{i}\circ Y_{i}-\left(P_{i}\circ X_{i}+E_{i}^{k}\right)+\frac{R_{i}^{k}}{\beta^{k}}∥}_{F}^{2}$$

Similarly, the subproblem in Equation (24) can be approximated as

(26) $$\begin{array}{cc} E_{i}^{k+1} & =arg\underset{E_{i}}{min}\lambda {∥E_{i}∥}_{F}^{2}+\frac{\beta^{k}}{2}{∥P_{i}\circ Y_{i}-\left(P_{i}\circ X_{i}+E_{i}^{k}\right)+\frac{R_{i}^{k}}{\beta^{k}}∥}_{F}^{2}\\ & =arg\underset{E_{i}}{min}\lambda Tr\left(E_{i}^{T}E_{i}\right)+\frac{\beta^{k}}{2}Tr\left(Q^{T}Q\right) \end{array}$$

where $Q=P_{i}\circ Y_{i}-\left(P_{i}\circ X_{i}+E_{i}^{k}\right)+\frac{R_{i}^{k}}{\beta^{k}}$. Equation (25) can be resolved by the linearized alternating direction method with an adaptive penalty [29] and a singular value thresholding operator [40], which have proven to be convergent. Taking the derivative of Equation (26) with respect to $E_{i}$ and setting it to zero, we can obtain

(27) $$E_{i}^{k+1}=R_{i}^{k}/\left(2\lambda +\beta^{k}\right)$$

When $X_{i}$ and $E_{i}$ are fixed, the update can be denoted:

(28) $$R_{i}^{k+1}=R_{i}^{k}+\beta^{k}\left(P_{i}\circ Y_{i}^{k+1}-\left(P_{i}\circ X_{i}^{k+1}+E_{i}^{k+1}\right)\right)$$

The penalty parameter β is updated by an adaptive strategy:

(29) $$\beta^{k+1}=min\left(\beta_{max},\rho \beta^{k}\right)$$

where $\beta_{max}$ is an upper bound of $\beta^{k}$ and ρ≥1 is a constant. Equation (27) has a closed solution which guarantees the convergence of the WGLRR method.

When we have estimated each group matrix with Equation (20) , the noise-free patches can be obtained by rearranging column vectors of each recovered group matrix. Considering that we take the *p*-most similar patches of $y_{i}$ to construct the group data matrix and each single patch might belong to several groups, we aggregate different estimates of this patch to obtain the noise-free image by a weighted averaging process. For the *i*th recovered group data matrix ${\hat{X}}_{i}$, the smaller the rank is, the higher the degree of linear correlation the patches have. Thus, the estimation of pixels in this group data matrix is better. We average different estimates of the pixel *i* with different weights to suppress noise further.

(30) $$x_{i}=\frac{\sum_{j}w_{j}x_{i,j}}{\sum_{j}w_{j}}$$

where $x_{i,j}$ is the *j*th recovered version of the pixel *i* and $w_{j}$ is the rank-related weight of the *j*th group matrix defined as

(31) $$w_{j}=\left\{\begin{array}{c} 1-\frac{k}{p+1},k<p+1\\ \frac{1}{p+1},k=p+1 \end{array}\right.$$

where *k* is the rank of the *j*th recovered group data matrix. The weighted averaging procedure ensures that the better recovered results of a pixel in different groups will contribute more to the final denoised version $x_{i}$.

### 3.3. Boosting of the Image Denoising Method

Despite the effectiveness of the above denoising method, improved results can be obtained by applying a boosting technique. On the one hand, signal leftovers and noise leftovers reside in the residual image. On the other hand, the gap between the local patch-processing and the global need for a whole restored image is a major shortcoming in the patch-based methods. Thus, we perform a boosting strategy to improve the results which strengthens the signal by leveraging the availability of the denoised image. The improved denoising performance is gained by applying the WGLRR as a black-box. The first step is to strengthen the signal by adding the previous denoised image to the noisy input image. The second step is to operate the denoising method on the strengthened image. The third step is to add the method noise and normalization. The procedure can be denoted by the core equation:

(32) $${\hat{X}}^{l+1}=\frac{1}{1+\gamma }\left[f\left(Y+\gamma {\hat{X}}^{l}\right)+\left(Y-f\left(Y\right)\right)\right]$$

where ${\hat{X}}^{0}=0$. The parameter γ controls the signal emphasis and a large value of γ implies a strong emphasis of the underlying signal. Because the estimated part of the signal is emphasized, there is no loss of signal content that has not been estimated correctly. The literature [35] has proven that the signal strengthened image can be denoised more effectively compared to the noisy input image.

The whole denoising algorithm is summarized in Algorithm 1. The detail of WGLRR can be seen in Algorithm 2.

**Algorithm 1:** The proposed SAR image despeckling method.Input: the noisy SAR image Y, the parameter γ
  Initialize
${\hat{X}}^{\left(0\right)}=0,\;Y^{\left(0\right)}=Y$
  for l = 1:T do
    Iterative regularization $Y^{\left(l\right)}=Y+\gamma {\hat{X}}^{\left(l-1\right)}$
    for each patch $y_{i}$ in $Y^{\left(l\right)}$
do
      Group data matrix $Y_{i}\in {ℜ}^{M\times (p+1)}$
      Despeckle via WGLRR       Obtain the denoised version $X_{i}^{k+1}$
    endfor
    Aggregate $X_{i}$ to form the clean image ${\hat{X}}^{\left(l\right)}$
    ${\hat{X}}^{l}=({\hat{X}}^{l}+\left(Y-f\left(Y\right)\right)/\left(1+\gamma \right)$
  endfor Output: Clean image ${\hat{X}}^{l}$


**Algorithm 2:** WGLRR.  Input: Group data matrix $Y_{i}$
    Initialize
$X_{i}^{0}=0,\;E_{i}^{0}=0,\;R_{i}^{0}=0,\;\beta^{0}=0.1,\;\beta_{\max}=10^{10},\;\lambda =0.1$
    Repeat
      Update $X_{i}^{k+1}$ using Equation (25)       Update $E_{i}^{k+1}$ using Equation (26)       Update $R_{i}^{k+1}$ using Equation (28)       Update $\beta^{k+1}$ using Equation (29)       k=k+1
    enduntilconvergence  Output:
$X_{i}^{k+1}$

## 4. Experimental Results and Analysis

To demonstrate the efficiency of our method, we compare it with other state-of-the-art speckle removal algorithms, such as the Frost filter (Frost) [6], the original non-local weighted group low-rank representation (WGLRR) [31], the blind de-noising algorithm based on weighted nuclear norm (BWNNM) [32], K-SVD [17] and the nonlocal fast adaptive nonlocal SAR de-noising (FANS) [25]. All experiments were carried out by Matlab (MathWorks, Natick, MA, USA) codes on Intel Core i5 3.1 GHz (Intel Corporation, Santa Clara, CA, USA) with 4 GB RAM. Due to the lack of an ideal noiseless image, it is a challenge to make an objective assessment. From the literature review [14,15,25], we performed experiments on simulated images to analyze the objective assessment comparing with different denoising methods. In Section 4.2, we discuss experiments with actual SAR images.

### 4.1. Results with Simulated Images

**Figure 3:**
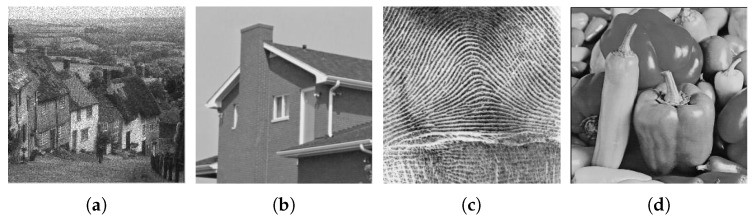
Test speckle free images: (**a**) Hill; (**b**) House; (**c**) Fingerprint; and (**d**) Peppers.

In this section, we multiply four optical images by simulated white speckle in amplitude format with pdfs corresponding to the cases of 1, 2, 4, 8 and 16 looks. The original optical images are shown in Figure 3. We evaluate and compare the different methods by two objective valuation index measures: peak signal-to-noise-ratio (PSNR) and structural similarity (SSIM). PSNR can measure the intensity difference between two images and SSIM can better reflect the structure similarity between the target image and the reference image. PSNR can be formulated as follows.

(33) $$PSNR=10log_{10}\frac{{\left|x\right|}_{max}^{2}}{MSE}$$

where

(34) $$MSE=\langle {\left[x\left(i\right)-\overset{\wedge }{x}\left(i\right)\right]}^{2}\rangle$$

and ${\left|x\right|}_{max}$ is the maximum value admitted by the data format. The mean-square error (MSE) is computed as a spatial average $\langle \cdot \rangle$, with *x* and $\hat{x}$ being the original and denoised images, respectively. SSIM can be calculated by Equation (35).

(35) $$SSIM=\frac{2\left(\mu_{i}\mu_{j}+C_{1}\right)\left(2\sigma_{ij}+C_{2}\right)}{\left(\mu_{i}^{2}+\mu_{j}^{2}+C_{1}\right)\left(\sigma_{i}^{2}+\sigma_{j}^{2}+C_{2}\right)}$$

where μ and $\sigma^{2}$ denote the mean value and variance, respectively; *i* and *j* denote different areas; and $C_{1}$ and $C_{2}$ are the constant with small value to ensure the denominator is not zero. We average the values of PSNR and SSIM from the four test images and report the results of different methods in Table 1. In Table 1, we can see that the Frost filter improves several decibels with respect to the noisy images with different looks. However, the more sophisticated methods proposed recently have a higher PSNR. The original WGLRR method is improved by about 2–4 dB compared to Frost, especially in the case of L=16. This is because, with the increase of the looks, the speckle model is similar to obeying Gauss distribution which is more suitable for WGLRR. The K-SVD method obtains a higher PSNR but lower SSIM. The proposed method achieves a PSNR gain of 1–3 dB compared to WGLRR and BWNNM and is similar PSNR to FANS. Meanwhile, the SSIMs in the proposed method are the closest to 1 which indicates that the denoised results by our method are the most similar to the original images.

**Figure 4:**
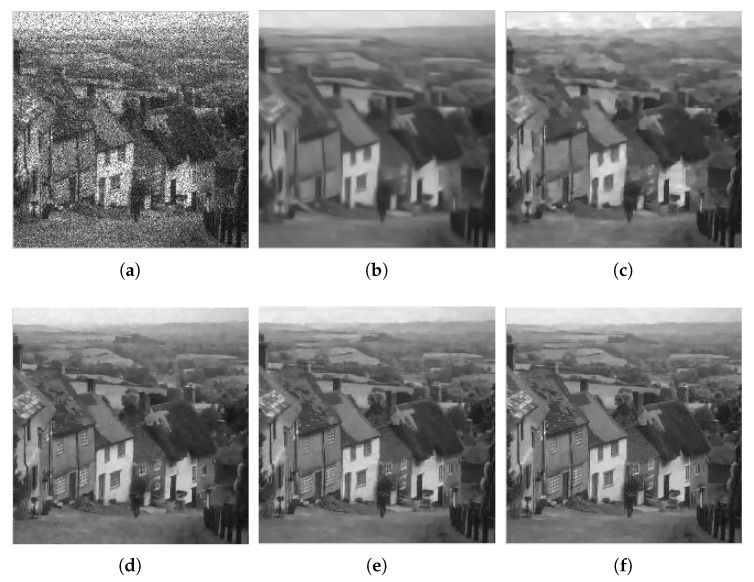
Filtered images for Hill corrupted by four-look speckle: (**a**) Frost; (**b**) WGLRR; (**c**) BWNNM; (**d**) K-SVD; (**e**) FANS; and (**f**) the proposed method.

**Figure 5:**
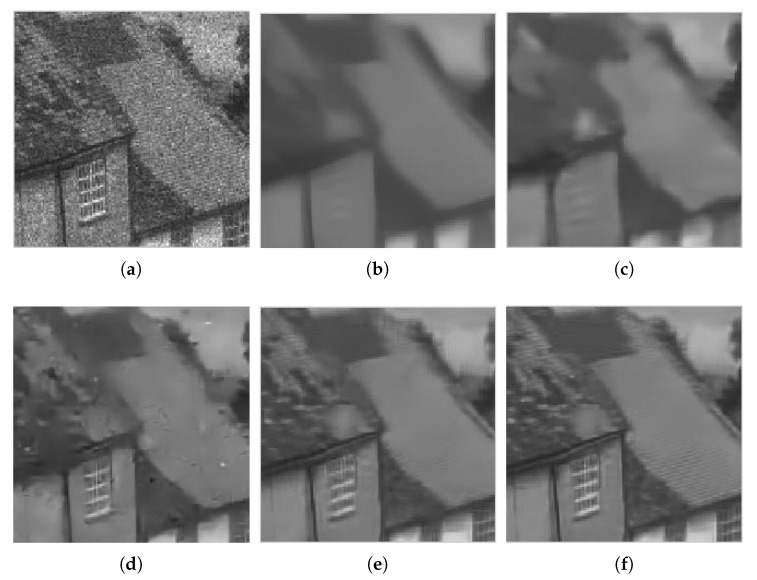
Zoom of filtered images for Hill corrupted by four-look speckle: (**a**) Frost; (**b**) WGLRR; (**c**) BWNNM; (**d**) K-SVD; (**e**) FANS; and (**f**) the proposed method.

Due to the limitation of space, we only show the denoised images provided by all methods for Hill with L=4 in Figure 4. Figure 5 shows the zoom of the roof area in the Hill image. Although the Frost filter can reduce the noise to some extent, the denoised image still has room for noise reduction. Both the original WGLRR and BWNNM methods are verified for good superiority in terms of image denoising at the cost of a certain amount of boundary blurring. The K-SVD method blurs the details and has some artifacts. FANS and the proposed method can reduce the noise while reserving the detail feature. In Figure 5, we can see that the roof tiles and the window become clearer with the proposed method.

**Figure 6:**
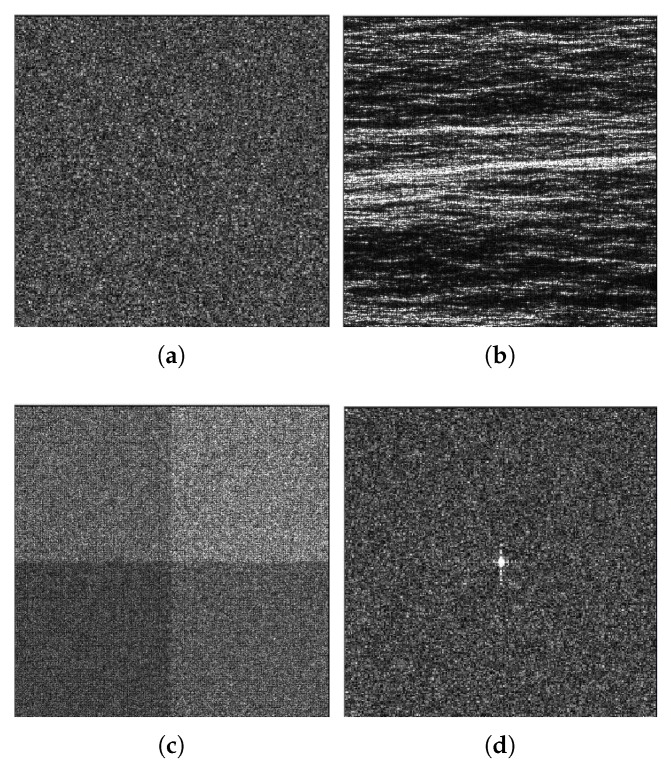
Images of the benchmark: (**a**) Homogeneous; (**b**) DEM; (**c**) Squares; and (**d**) Corner.

To gain better insight into the advantage of our method, Table 2 shows the objective indicators [41] on the single-look SAR images of Figure 6, generated by physical-level simulation. In the homogeneous region, ENL is computed to measure the speckle suppression of different methods. In the digital elevation model (DEM), the coefficient of variation (Cx) accounts for the texture preservation. In the squares, we use edge smearing (ES) to measure edge profile degradation. In the corner region, the contrast to background (Cbg) accounts for the radiometric fidelity. In Table 2, we can see that the K-SVD has the largest ENL, implying the strongest speckle suppression. FANS and our method guarantee a better preservation of features in textured areas with Cx > 2.2. The best edge smearing is not to filter at all while our method has the same competitiveness as FANS. From the last column, we can see that our method has higher Cbg than the other methods which shows a good radiometric accuracy.

### 4.2. Results with Actual Synthetic Aperture Radar Images

**Figure 7:**
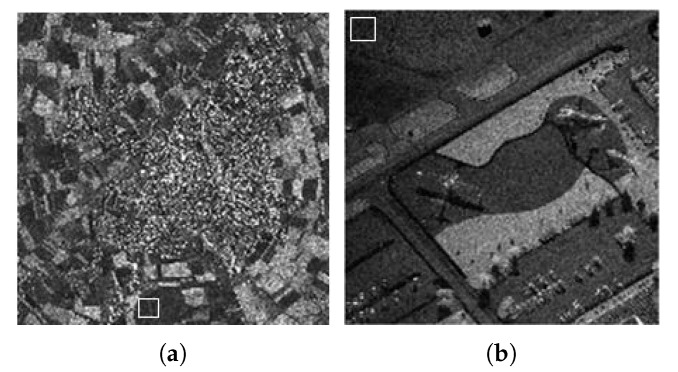
Test SAR images with selected areas for ENL computation (white box): (**a**) MR; and (**b**) CB.

**Figure 8:**
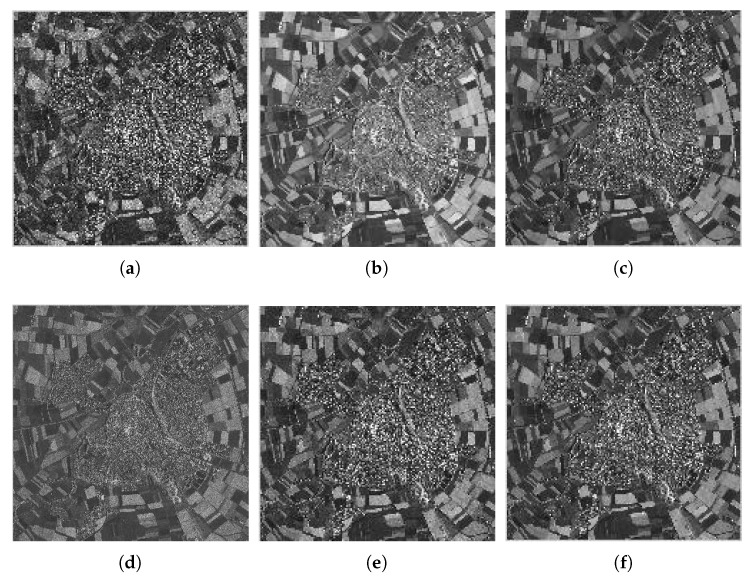
Filtered images for MR: (**a**) Frost; (**b**) WGLRR; (**c**) BWNNM; (**d**) K-SVD; (**e**) FANS; and (**f**) the proposed method.

**Figure 9:**
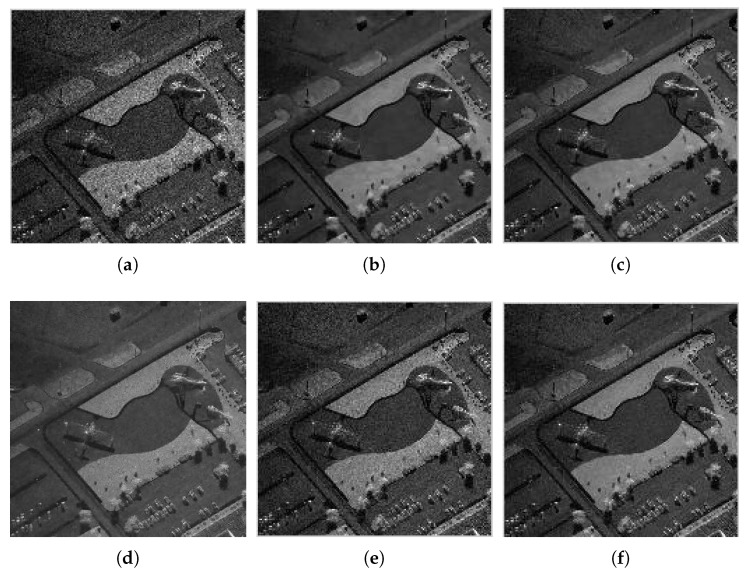
Filtered images for CB: (**a**) Frost; (**b**) WGLRR; (**c**) BWNNM; (**d**) K-SVD; (**e**) FANS; and (**f**) the proposed method.

To verify the practicability of the proposed method, we also need to examine the proposed method in the actual SAR image. They are, a Terra-SAR image with 1-m resolution Meteorite (MR) scene of the Swabian Jura and a mini-SAR image with 4 in resolution Capitol Building (CB) scene, courtesy of Sandia National Laboratory (Figure 7). Figure 8 and Figure 9 show the filtered images. For these images, we compute the ENL and computation time (Time). Larger ENL values indicate stronger speckle rejection and a higher ability to distinguish different gray levels. We measure the homogeneous area with white boxes in Figure 7. Table 3 shows the ENL values and the computation time for different methods. It can be seen in Table 3 that WGLRR has the biggest ENL values than the other methods which indicates the strongest speckle rejection ability. However, the strong noise reduction comes at the cost of some loss of details.

**Figure 10:**
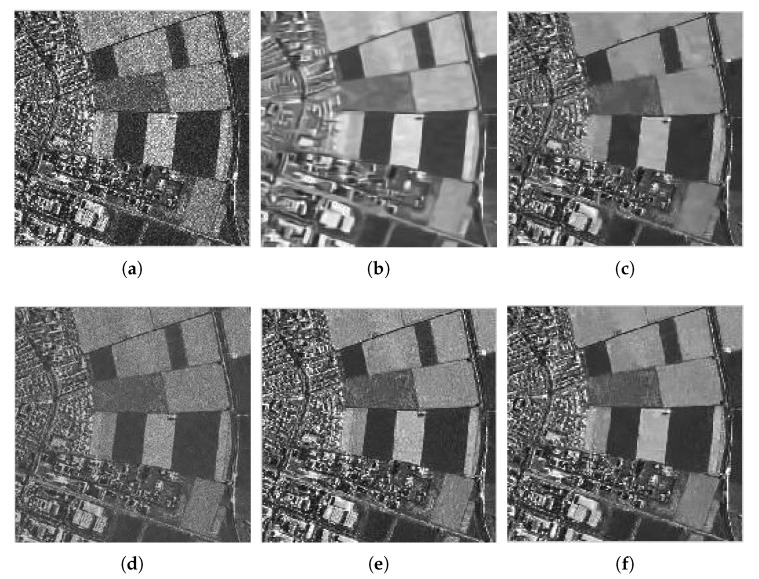
Zoom of filtered images for MR: (**a**) Frost; (**b**) WGLRR; (**c**) BWNNM; (**d**) K-SVD; (**e**) FANS; and (**f**) the proposed method.

**Figure 11:**
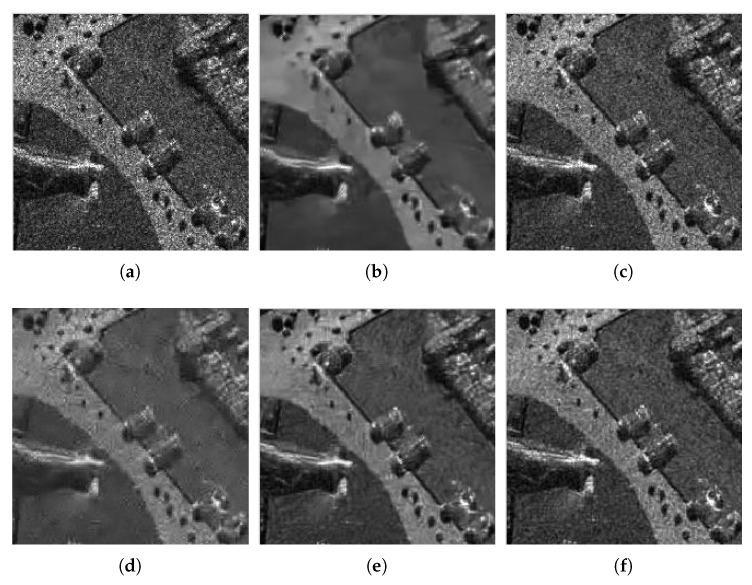
Zoom of filtered images for CB: (**a**) Frost; (**b**) WGLRR; (**c**) BWNNM; (**d**) K-SVD; (**e**) FANS; and (**f**) the proposed method.

**Figure 12:**
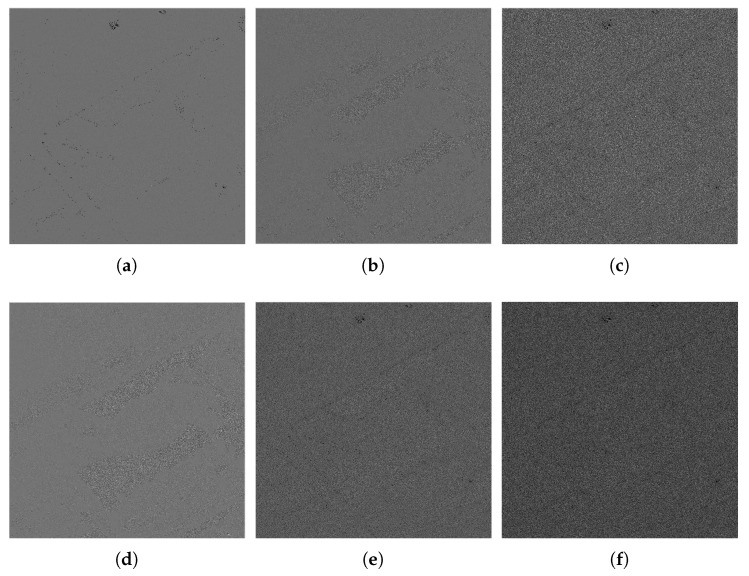
Ratio images of different denoised methods for CB: (**a**) Frost; (**b**) WGLRR; (**c**) BWNNM; (**d**) K-SVD; (**e**) FANS; and (**f**) the proposed method.

To achieve a clearer display, we also select parts of the denoised images to amplify, as shown by Figure 10 and Figure 11. We can see that, although the ENLs of WGLRR and K-SVD are superior to our method, they oversmooth the image edges and details. As an excellent denoising algorithm, the FANS filter combines the nonlocal filtering approach with other effective denoising tools which can retain the image details and textures while improving speckle suppression effectively. However, some typical wavelet artifacts appear because of the wavelet shrinkage, as shown in Figure 10e and Figure 11e. Our method seems to be the best tradeoff between protecting the details and reducing the noise, while avoiding the artifacts effectively. As a kind of non-reference measure, the visual inspection of the ratio image can provide not only information on the ability of edge-preservation but also indications of filtering artifacts [41]. Due to limited space, we only show the ratio image of Figure 7b by different denoising methods in Figure 12. In Figure 12, we can see that the ratio image of our method has the least edge information and is close to speckle. Considering the ratio statistics, on the one hand, the mean value of the ratio image is usually used to test the level of bias. Since the denoised methods are designed to preserve the mean of backscattered intensity, the mean value should be equal to one [24]. The methods involved in this paper shows from a minimum of about 0.77 for WGLRR, through 0.87 for FANS and 0.89 for the proposed method, to a maximum of 0.92 for BWNNM which indicates that the proposed method has the smaller bias. On the other hand, the ENL of the ratio images indicates the speckle power suppression. The pixels in the white box of Figure 7 are used to compute the ENL of the ratio image (ENLr). The average ENLr of the proposed method is about 2.97 and much close to the same of the original images which also reflects the stronger speckle suppression ability. According to the above analysis, our method guarantees a significant noise reduction without introducing some kinds of artifacts and may be a promising despeckling method.

## 5. Conclusions

We proposed a boosting SAR despeckling method based on the non-local weighted group low-rank representation. Taking into account the probabilistic noise distribution of speckle in SAR images, we used the ad hoc block similar measure to form a data matrix. Integrating the corrupted probability of each pixel to the LRR model, we were able to constrain the fidelity of the recovered image. Using a weighted averaging procedure to suppress noise and boosting the algorithm to reduce the local-global gap, we were able to obtain the denoised image. Experimental results verify the validity of the proposed method. The edges and structures in the SAR images can be well reserved with fewer artifacts, which is very important for the interpretation of other SAR images. Compared with other methods tested in our paper, our method contains the block similarity measure step in the nonlocal area, singular value decomposition in the SVT operator and boosting of the method which are very time consuming. In future work, we will seek a new computation to reduce the complexity to apply the proposed method to SAR images, achieving superior despeckling.

## Figures and Tables

**Table 1:** The average peak signal-to-noise-ratio (PSNR) (dB) and structural similarity (SSIM) results of the denoised image by different methods.

	L = 1		L = 2		L = 4		L = 8		L = 16	
	**PSNR**	**SSIM**	**PSNR**	**SSIM**	**PSNR**	**SSIM**	**PSNR**	**SSIM**	**PSNR**	**SSIM**
**Noisy**	11.72	0.1680	14.49	0.2556	17.40	0.3598	20.38	0.4743	23.37	0.5917
**Frost**	17.68	0.5891	20.53	0.6274	24.45	0.6990	25.89	0.7241	26.98	0.7829
**WGLRR**	19.89	0.6215	22.78	0.6565	27.79	0.7287	28.03	0.7811	30.96	0.8305
**BWNNM**	19.87	0.6220	26.80	0.6590	28.81	0.7302	28.89	0.7976	30.98	0.8590
**K-SVD**	23.08	0.6341	29.21	0.6870	31.76	0.7450	34.08	0.8077	34.01	0.8721
**FANS**	20.02	0.7565	28.90	0.7981	29.98	0.8341	30.02	0.8672	32.87	0.9061
**Proposed method**	20.04	0.7587	28.92	0.7992	30.06	0.8451	33.21	0.8892	33.90	0.9221

**Table 2:** The objective indicators of the single-look SAR images on the despeckling benchmark.

	ENL	Cx	ES	Cbg
	**Homogeneous**	**DEM**	**Squares**	**Corner**
**Noisy**	1.0	3.54	0.029	36.50
**Frost**	17.8	1.98	0.138	36.41
**WGLRR**	200.3	1.82	0.201	30.62
**BWNNM**	123.8	2.03	0.278	32.80
**K-SVD**	231.7	2.09	0.231	33.89
**FANS**	161.1	2.55	0.155	35.50
**Proposed method**	180.6	2.23	0.189	35.65

**Table 3:** ENL and computation time for actual SAR images.

	MR		CB	
	**ENL**	**Time (s)**	**ENL**	**Time (s)**
**Noisy**	3.01	—-	2.99	—-
**Frost**	7.98	80.31	8.02	76.18
**WGLRR**	101.6	160.1314	235.2	158.9082
**BWNNM**	87.9	156.7239	92.6	148.0935
**K-SVD**	100.3	280.4576	250.9	340.9642
**FANS**	20.5	135.4590	36.3	132.5261
**Proposed method**	39.2	207.8261	63.6	198.9801
